# Preoperative morbidity and joint awareness while awaiting hip arthroscopy for femoroacetabular impingement

**DOI:** 10.1186/s40634-021-00431-1

**Published:** 2021-12-04

**Authors:** P. G. Robinson, T. R. Williamson, I. R. Murray, J. F. Maempel, D. J. MacDonald, D. F. Hamilton, P. Gaston

**Affiliations:** 1 Edinburgh Orthopaedics, Royal Infirmary of Edinburgh, Edinburgh, UK; 2 Edinburgh Medical School, The University of Edinburgh, 47 Little France Crescent, Edinburgh, EH16 4TJ UK; 3 Department of Orthopaedic Surgery, Stanford University, Redwood, California USA; 4 Department of Trauma & Orthopaedics, Royal Prince Alfred Hospital, Camperdown, New South Wales Australia; 5 School of Health and Social Care, Edinburgh Napier University, Edinburgh, UK

**Keywords:** Femoroacetabular, Impingement, Hip, Awareness, FJS-12

## Abstract

**Purpose:**

The Forgotten Joint Score (FJS-12) is a valid tool in the evaluation of patients undergoing hip arthroscopy, assessing the unique concept of joint awareness in the setting of a patient’s hip pathology. The preoperative burden on patients’ mental wellbeing of impaired joint function or symptoms is well established. The purpose of this study was to determine patients’ awareness of their hip joint whilst awaiting hip arthroscopy for femoroacetabular impingement, to explore any association between joint awareness and mental health status, and to determine whether this relates to time spent waiting for arthroscopy preoperatively.

**Methods:**

A prospective database of patients undergoing hip arthroscopy between January 2018 and November 2020 was analysed. All patients with a diagnosis of femoroacetabular impingement (FAI) undergoing arthroscopic treatment were included. Questionnaires included the FJS-12, twelve item international hip outcome tool (iHOT-12), EuroQol 5D-5L (EQ-5D-5L) and the Tegner activity score. Pearson’s correlation coefficient was used to assess relationships between continuous variables.

**Results:**

Preoperative functional outcomes were completed by 81 patients (97.5%) prior to undergoing hip arthroscopy. Median preoperative FJS-12 score was 16.67 (IQR 8.33 – 29.68). Forty-four patients reported any level of anxiety/depression preoperatively (54.3%). Preoperative FJS-12 showed a significant negative correlation with worsening mental health status (r = − 0.359, *p* <  0.001), and a significant positive correlation with EQ-5D-5L (r = 0.445, *p* <  0.001). The duration of symptoms or time on the waiting list did not correlate with increased joint awareness or worsened mental health.

**Conclusion:**

Joint awareness is high when awaiting hip arthroscopy for FAI. Increasing levels of joint awareness correlate with poorer mental health status and poorer quality of life measures, however these parameters do not seem to be associated with increased duration of symptoms prior to surgery or time on the waiting list for surgery.

## Background

Hip arthroscopy has been shown to provide superior outcomes for femoroacetabular impingement (FAI) compared to physiotherapy alone [14, 27]. Many patient reported outcome measures (PROMs) have been designed to detect symptoms related to non-arthritic hip problems [9, 15, 23, 26, 37]. Behrend et al introduced the concept of joint awareness to hip and knee arthroplasty with good effect with the Forgotten Joint Score (FJS-12) [2] which has been reported to achieve distinction between high performing older patients, which previous PROMs may have struggled to do [16]. Joint awareness is distinct from joint function or symptomatic state, and reflects a host of interplaying factors that impact on the ability of a patient to ‘forget’ their joint pathology or replacement, and has its own distinct biological basis [17, 22]. This concept is well suited for capturing reliable and valid outcomes for young, active patients undergoing hip arthroscopy [3, 28].

Impaired joint function and increased symptom burden preoperatively have been shown to be associated with increasing levels of anxiety and depression in patients with FAI [18], and the presence and severity of depression have been shown to be predictive of poor functional outcomes following hip arthroscopy [31, 33]. Furthermore, Stone et al. reported that those with minimal depressive symptoms were more likely to achieve the substantial clinical benefit and the patient acceptable symptomatic score compared to those with moderate to severe symptoms at 1 year follow up [31]. Whilst the association between joint function or symptomatology and worse mental health status is established, it has not yet been determined whether the same is true for a patient’s awareness of their hip joint. Waiting times for elective orthopaedic surgery in the United Kingdom are now at a record high [6]. With a growing number of patients waiting for surgery, it is important that hip arthroscopists are conscious of the preoperative morbidity of femoroacetabular impingement and the effect this has on patients’ mental health.

The purpose of this study was to assess the preoperative burden of joint awareness on patients undergoing hip arthroscopy for FAI, to establish whether there is a relationship between joint awareness and mental health status for these patients, and to determine whether this relates to time spent waiting for arthroscopy preoperatively.

## Patients and methods

**Fig. 1:**
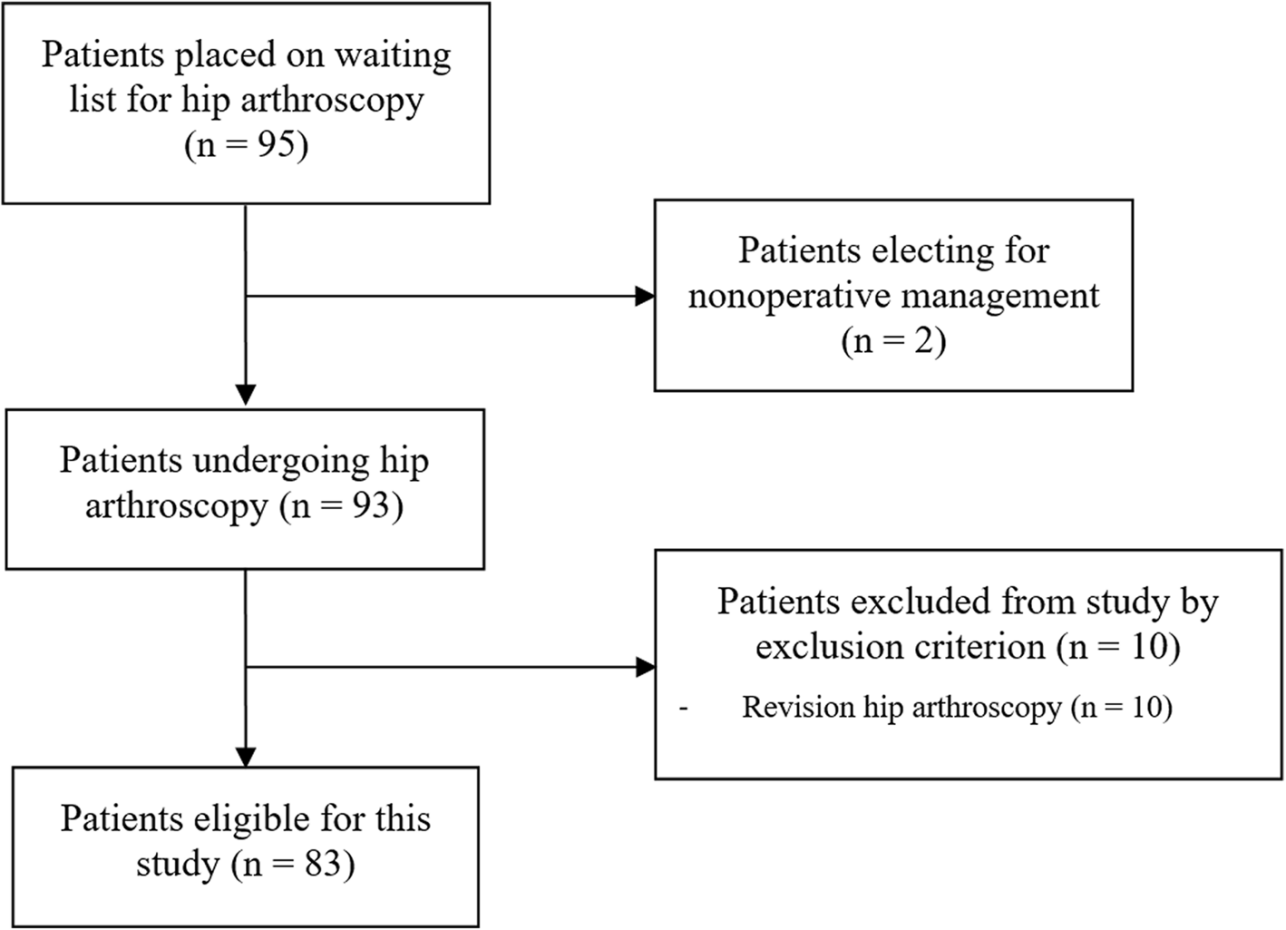
Flowchart of patient inclusion and exclusion in this study

Patients for this study were identified from a prospective database of patients placed on the waiting list for hip arthroscopy by a single surgeon within the date range January 2018 to November 2020. All patients diagnosed with FAI and labral tears during this time frame were included. Exclusion criteria were patients undergoing revision arthroscopy, or those with a Tönnis grade of > 1 (Fig. 1). The treating surgeon had diagnosed all patients in this study with FAI. Diagnoses were made as per the Warwick Agreement consensus using a combination of clinical history, examination, plain radiographs, and magnetic resonance arthrogram if appropriate [13]. Patients had failed a trial of non-operative treatment including analgesia and physiotherapy. In cases of doubt, intra-articular injections were used to confirm the origin of symptoms. Patients completed preoperative functional questionnaires 2 weeks prior to surgery at the pre-assessment clinic.

### Outcome measures

Demographic data included age, sex, body mass index (BMI), smoking status and Scottish Index of Multiple Deprivation (SIMD) vigintile (a relative measure of deprivation) [36]. Preoperative patient reported outcomes measures collected included: the 12 item international hip outcome tool (iHOT-12) [15], FJS-12 [2], EuroQol-five Dimensions-5 L (EQ-5D-5L) [32] and Tegner activity grading scale [35]. The FJS-12 contains 12 questions which are scored using a Likert scale ranging from 0 to 4. The total sum score is converted into a scale ranging from 0 to 100, with lower scores reflecting more joint awareness during activities of daily living [2]. Mental health was assessed using the anxiety/depression subdomain of the EQ-5D-5L score. This has previously been shown to be a valid and reliable tool for assessing overall mental health [4, 5, 11]. Patients completed a five point Likert scale with options including “I am not anxious or depressed”, “I am slightly anxious or depressed”, “I am moderately anxious or depressed”, “I am severely anxious or depressed”, “I am extremely anxious or depressed” [32]. Pain was scored on a visual analogue scale between 0 to 10 where a rating of 10 was the worst pain patients had experienced.

Self-reported duration of symptoms and time on the waiting list were recorded in months. For duration of symptoms patients were asked “How long have you been experiencing the symptoms in your hip that caused you to seek medical advice?”. Data pertaining to previous contralateral FAI surgery and revision surgery was collected. Morphological hip data included the presence of cam or pincer lesions, centre-edge angle (CEA) and Tönnis grading. Cam deformity was classified as an alpha-angle of greater than 60^o^ [1].

### Statistical analysis

Statistical analysis was undertaken using Statistical Package for Social Sciences (SPSS) software (IBM, Inc., Armonk, New York, United States) v24. Normality was assessed using Shapiro-Wilk testing. Non-parametric data was reported as median with interquartile range and compared using Mann Whitney U-tests, Wilcoxon signed ranks tests and Kruskal-Wallis tests. Cross-tabulated data for dichotomous variables were analysed using chi squared tests. Correlation of continuous variables was assessed using Pearson correlation coefficient and ordinal variables were assessed using Spearman rank test. A correlation coefficient for each test greater than 0.6 was considered strong, 0.4 to 0.59 was considered moderate, 0.2 to 0.39 was considered weak and <  0.2 was considered very weak [34]. A *p*-value of < 0.05 was considered statistically significant.

## Results

**Fig. 2:**
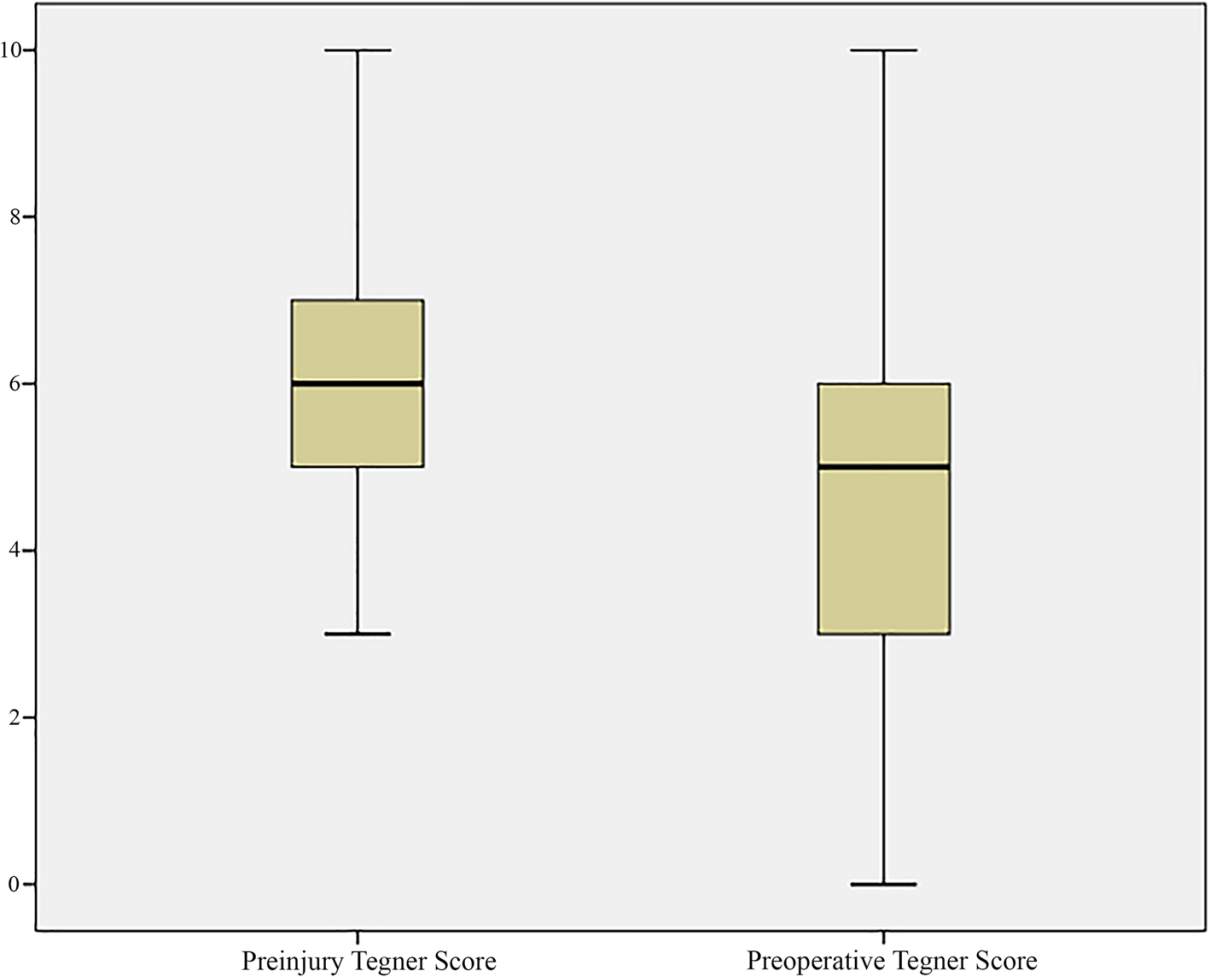
Pre-injury and preoperative Tegner activity scores

There were 83 patients who were eligible, and 81 completed preoperative PROMs scores during the study period. Median age at the time of assessment was 31 years (IQR 23 – 36) and the median BMI was 24.2 (IQR 22.1 – 28.1). Preoperative functional scores, and demographic and radiographic variables are presented (Table 1). Tegner scores decreased from 6 (IQR 5 – 7) prior to injury to 5 (IQR 3 - 6) prior to surgery (*p* <  0.001) (Fig. 2). Median self-reported duration of symptoms was 29 months (IQR 18.25 - 48) and time on the waiting list was 4.2 months (IQR 3.3 – 5.6; range 0.5 – 12.5). Nine patients (10.8%) had previously undergone arthroscopy on their contralateral hip.

**Table 1:** Demographic data, radiographic data and preoperative functional scores

	***n*** = 83
**Age**	31.0 (IQR 23.0 – 36.0)
**Gender**	53 female: 30 male
**BMI**	24.2 (IQR 22.1 – 28.1)
**SIMD Vigintile**	13 (IQR 9 – 17)
**Co-morbidities**	
**Asthma**	7
**Hypothyroidism**	6
**Spondylarthropathy**^a^	5
**Hypothyroidism**	2
**Diabetes**	1
**Smoker status**	
**Smoker**	8 (9.7%)
**Non-smoker**	75 (90.3%)
**Preoperative FJS-12**	16.67 (IQR 8.33 – 29.68)
**Preoperative iHOT-12**	37 (IQR 22 – 45.5)
**Preoperative EQ-5D-5L Index**	0.620 (IQR 0.35 – 0.703)
**Preoperative EQ-5D-5L VAS**	70 (IQR 60 – 80)
**Preoperative Tegner Score**	5 (IQR 3 – 6)
**Center edge angle**	37.24 (SD 8.68)
**Tönnis grade**	
**0**	39 (47.0%)
**1**	44 (53.0%)
**Hip morphology**	
**Cam**	12 (14.5%)
**Pincer**	3 (3.6%)
**Mixed**	68 (73.1%)

*BMI* Body mass index, *SIMD* Scottish Index of Multiple Deprivation, *iHOT-12* 12 item international hip outcome tool, *FJS-12* Forgotten Joint Score, *EQ-5D-5L* EuroQol-five Dimensions-5 L, *VAS* visual analogue scale; ^a^Two patients with ankylosing spondylitis, two with juvenile idiopathic arthritis and one patient was anti cyclic citrullinated peptide positive

### Forgotten joint score

Median preoperative FJS-12 score was 16.7 (IQR 8.3 – 29.7) (Table 1). FJS-12 score showed a significant positive correlation with EQ-5D-5L index (*p* <  0.001) and a significant negative correlation with anxiety/depression scores (*p* <  0.001) (Table 2). Previous surgery on the contralateral hip was not associated with significant variation in FJS-12 score (*p* = 0.408). FJS-12 scores were not statistically different according to smoking status (*p* = 0.355).

**Table 2:** Univariate correlation analysis for variables associated with the FJS-12

	r	***P***
**iHOT-12**	0.697	< 0.001
**EQ-5D-5L index**	0.445	< 0.001
**EQ-5D-5L VAS**	0.449	< 0.001
**Anxiety/depression score**	−0.359	< 0.001
**VAS pain score**	−0.401	< 0.001
**CEA**	0.059	0.601
**Tönnis grade**	−0.131	0.242
**BMI**	−0.237	0.032
**Age**	−0.245	0.027
**Preoperative symptom duration**	−0.04	0.749
**Days spent on waiting list**	−0.031	0.788

*iHOT-12* 12 item international hip outcome tool, *FJS-12* Forgotten Joint Score, *EQ-5D-5L* EuroQol-five Dimensions-5 L, *VAS* visual analogue scale, *BMI* Body mass index, *CEA* Centre-edge angle

### Mental health status

**Fig. 3:**
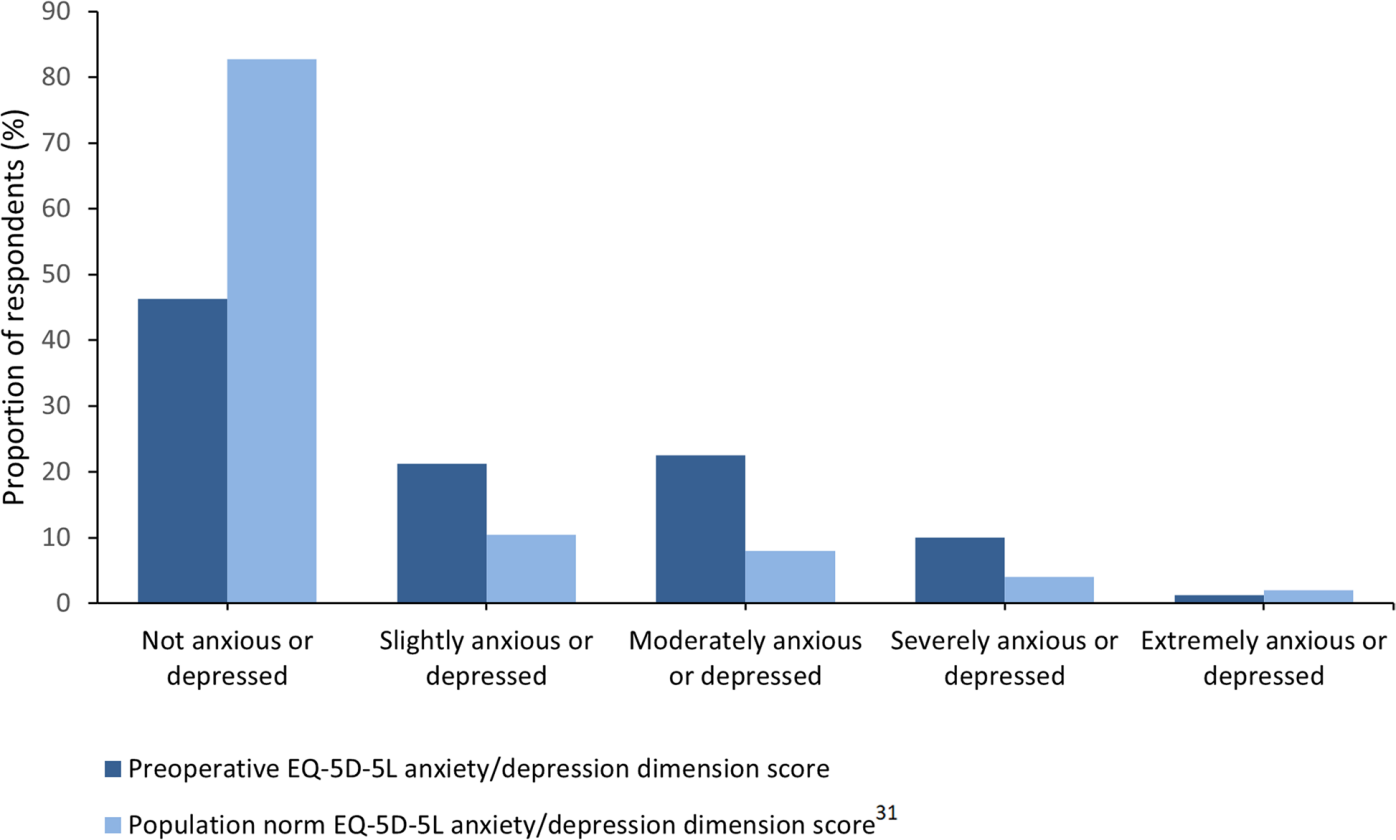
Degrees of anxiety/depression compared to population norms

Sub-domain analysis of the EQ-5D-5L can be seen in Table 3. Six patients had a formal diagnosis of anxiety and/or depression from their general practitioner at the time of the study. Forty-four patients (54.3%) reported at least slight anxiety/depression at the time of assessment (Fig. 3). There was a strong, negative correlation between increasing degrees of anxiety/depression and the overall EQ-5D-5L index (r = − 0.691, *p* <  0.001) and moderate negative correlations with EQ-5D-5L VAS (r = − 0.449, *p* <  0.001) and iHOT-12 scores (r = − 0.458, *p* <  0.001). There was a weak, negative correlation between increasing degrees of anxiety/depression and preoperative Tegner activity scores (r = − 0.307, *p* = 0.011). A weak correlation was seen between increasing anxiety/depression scores and both BMI (r = 0.328, *p* = 0.003) and smoking status (r = 0.331, *p* = 0.006). There was no significant correlation between increased degrees of anxiety/depression and age (r = − 0.011, *p* = 0.923), SIMD vigintile (r = − 0.151, *p* = 0.181), pain score (r = 0.125, *p* = 0.309), duration of symptoms (r = 0.137, *p* = 0.267) or time on the waiting list (r = 0.010, *p* = 0.932). Preoperative Tegner scores were also correlated to EQ-5D-5L index(r = 0.288, *p* = 0.017) and EQ-5D-5L VAS (r = 0.434, *p* <  0.001).

**Table 3:** EQ-5D-5L results by each domain

	No problems	Slight problems	Moderate problems	Severe problems	Unable to do usual activities
**Mobility**	17	27	25	10	1
**Self-care**	55	14	10	1	0
**Usual activities**	14	18	27	15	6
**Pain/Discomfort**	3	10	37	27	3
**Anxiety/Depression**	37	17	18	8	1

## Discussion

The most notable findings from this study were 1) the high level of joint awareness in patients awaiting arthroscopic hip surgery for FAI (median FJS-12 score 16.7), 2) the high proportion of mental anxiety/depression reporting in patients awaiting hip arthroscopy, with 54.3% of patients reporting at least slight anxiety/depression, and 3) that there was a relationship between these variables.

Data relating to the concept of joint awareness has only been reported twice in the hip arthroscopy literature - when it was validated for this patient population [3, 28]. It has already been shown in the arthroplasty literature that joint awareness and other common functional outcome scores, such as the Oxford Hip Score are not perfectly correlated with one another, as they measure different, but related, constructs [21]. In this study, although there was strong correlation between the FJS-12 and the iHOT-12, this was not absolute, similarly suggesting that the FJS-12 captures additional data on patient perspectives. The FJS-12 will indirectly assess function to some degree, which may account for its correlation with other PROMs, as pain, stiffness, or limitation in function due to joint pathology will inherently make the patient ‘aware’ of the joint in those instances. However, joint awareness is a biologically distinct concept, triggered by multiple interplaying factors at a cortical level [17, 22]. Joint awareness is not just limited to symptomatology and functionality, but also reflects other less tangible influences on patients’ joint-related quality of life, such as psychosocial influences and patients’ expectations [2]. Therefore, it is imperative that we better understand joint awareness and its implications in FAI patients.

Preoperative joint awareness in this study was considerable, with a median FJS-12 score of 16.7, in comparison to population normative values of 87.5 reported in the literature [12]. This study establishes a relationship between the impact of joint awareness and mental health, something that has, to the best of our understanding, not previously been reported. Increasing levels of preoperative joint awareness correlated with greater severity of anxiety/depression and poorer health related quality of life, suggesting that awareness of a pathological hip is associated with worse self-reported mental health status and health related quality of life. This may be due to the FJS-12 capturing a more holistic approach to joint evaluation, assessing not just the symptomatic burden of pain, stiffness and function in activities of daily living, but also allowing for the psychosocial factors that affect patients’ wellbeing [2]. Additionally, general chronic disease states seen in wider clinical practice have well-established links with anxiety and depression [8, 39], and the functional limitations often resulting from such conditions can result in negative affective responses from the patient [38]. High levels of joint awareness as assessed by the FJS-12 reflect frequent awareness of the hip in a variety of activities, and so in the preoperative setting for FAI this may further highlight to the patient the chronicity of their symptoms and disability, in a similar vein to the day-to-day impact of chronic disease states on patients’ mental health status. Furthermore, the FJS-12 also captures patients’ awareness of their functional limitations, and restrictions in these may also correspond to poorer mental health [38].

There were high levels of self-reported anxiety/depression reported in this study and a previous study has reported inferior functional outcomes following hip arthroscopy in patients with significant depressive symptoms [31]. Age-matched population norms in the United Kingdom (UK) for the anxiety/depression domain of the EQ-5D-5L highlight the symptom burden in our patient cohort. Typically, 83% report no mental health symptoms and 17% of the population have at least slight anxiety/depression, whilst in our cohort 54.3% of patients had at least slight anxiety/depression [10]. Whilst there is little literature assessing the psychological burden of hip arthroscopy patients, high levels of anxiety/depression have been observed in other patient cohorts with femoroacetabular impingement, although comparisons between studies assessing mental health burden are limited by the heterogeneity of outcomes measures used [29, 31].

Scott et al reported the poor quality of life (QoL) status of patients while on the waiting list for joint replacement. The authors reported a median EQ-5D-5L index lower than that reported in this study (0.364 vs 0.584) and found 19% of patients [30] to be in a state ‘worse than death’ represented by a negative EQ-5D-5L index score [19, 30]. We found there to be 3.6% of patients with an EQ-5D-5L index score less than zero – or ‘worse than death’, and the median EQ-5D-5L index score in this study was well below the population norm for this age group (0.620 vs 0.939) [20]. This is especially pertinent given lengthy nature of elective surgical waiting lists [7], as the high levels of anxiety/depression and the poorer quality of life preoperatively are perhaps an overlooked burden on patients’ preoperative wellbeing whilst awaiting orthopaedic surgery, and one that surgeons should be aware of. Interestingly, neither prolonged symptom duration nor time spent on the elective surgical waiting list were correlated with patients’ anxiety/depression scores, suggesting that whilst patients’ preoperative mental health burden is considerable, it may not significantly deteriorate with increasing wait time preoperatively. However, further research assessing mental health status at multiple preoperative time-points would be required to definitively establish this.

This study is not without limitations. Retrospective self-reporting of symptom duration may lead to reporting bias and inaccuracies however recent studies have used this method reliably [25] and patients’ reporting of symptoms has been recommended over clinicians’ ratings [24]. The nature of presentation to our system precluded the ability to collect a prospective symptom diary. The definition of what constitutes a FAI-related symptom can be debated, however we took a pragmatic approach to this, asking patients how long the symptoms which caused referral to an orthopaedic surgeon, had been present. Though strongly correlated, one must resist the temptation to conclude that the symptoms associated with FAI were causative of anxiety or depression symptoms.

## Conclusion

Joint awareness is high when awaiting hip arthroscopy for FAI. Increasing levels of joint awareness correlate with poorer mental health status and poorer quality of life measures, however these parameters do not seem to be associated with increased duration of symptoms prior to surgery or time on the waiting list for surgery.

## Data Availability

The datasets used and/or analysed during the current study are available from the corresponding author on reasonable request.
